# Ionic liquids vs. ethanol as extraction media of algicidal compounds from mango processing waste

**DOI:** 10.3389/fchem.2022.986987

**Published:** 2022-09-16

**Authors:** Mateus L. Segatto, Lena Schnarr, Oliver Olsson, Klaus Kümmerer, Vania G. Zuin

**Affiliations:** ^1^ Department of Chemistry, Federal University of São Carlos, São Carlos, Brazil; ^2^ Institute of Sustainable Chemistry, Leuphana University of Lüneburg, Lüneburg, Germany; ^3^ Research and Education, International Sustainable Chemistry Collaborative Centre (ISC3), Leuphana University of Lüneburg, Lüneburg, Germany; ^4^ Green Chemistry Centre of Excellence, University of York, York, United Kingdom

**Keywords:** ionic liquids, ethanol, extraction, natural products, algicide, flavonoid, xanthonoid

## Abstract

The race for environmentally-safe pesticides and biocides has been showing solutions ranging from pest-pathologic microorganisms to safer botanical extracts that can be incorporated in several formulations. Often linked to high biological activities, fruit residues can be recovered from food processing factories to obtain complex extracts enriched with several bioactive chemicals. Mango (*Mangifera indica*) fruits are processed into food products in high volumes across the globe and generate a consistent residue that contains, among others, the xanthonoid mangiferin and the flavonoid hyperoside. Both compounds have been linked to several pharmacological and pesticidal activities, although not yet studied for algicidal applications, a current concern specially for antifouling and harmful algae blooms control products. The challenge lies, however, not only on the degree of activity of the natural compounds, but also on the processes necessary to separate, isolate and formulate the bioactive compounds in order to obtain an effective final product. The solvent choice plays an important part regarding the selectivity of the separation and isolation of the main bioactive compounds from the solid waste matrix. Ethanolic mixtures in water have been consolidated recently as a promising extraction medium for flavonoids and xanthonoids, although hindered by solubility limitations. In this paper, aqueous solutions of ionic liquids (ILs) were tested, screened and optimized using Box-Behnken design and Response Surface Methodology to obtain mangiferin and hyperoside-enriched extracts. Results showed a greater concentration of mangiferin and hyperoside with 1-octyl-3-methylimidazolium chloride ([C_8_MIm] Cl), when compared to choline acetate and ethanolic extracts using optimized parameters. In terms of sufficiency, solvent selection between ILs and ethanolic extraction media was discussed considering economic and environmental factors. Ethanol/water mango waste extracts were then studied for their activity against *Raphidocelis subcapitata* microalgae, which showed a higher growth inhibition in comparison to standard solutions of mangiferin and hyperoside, either individually and in a 1:1 mixture. A EC_50_ value was found in relative low concentrations of mangiferin and hyperoside (0.015 mg L^−1^) detected in the extract, showcasing a promising approach to the direct use of residuary plant extracts in biocidal formulations.

## 1 Introduction

Synthetic pesticides used in crops, households and other applications have their negative impacts to earth systems well established in the last decades, especially in regards to water and soil pollution and biodiversity (Stehle and Schulz, 2015; Schulz et al., 2021). In the search for alternatives, among several novel pest management practices, the use of natural pesticides, also referred as biopesticides, stands out as a potentially sustainable approach to pest control. A potential source of natural biological active compounds is discarded as waste or used for low-value applications in agricultural fields and food processing plants across the globe (Matharu et al., 2016; Naumovski et al., 2017).

The fruits of mango (*Mangifera indica*) represent an opportunity for the production of bioactive compounds extracted from their discarded fractions. Led by India, which represents 44% of the world’s production, the harvest of mango fruits has reached nearly 55 million tons in 2020, almost exclusively in tropical countries, a number that has been consistently growing in recent years (FAO, 2022). The main by-products generated in mango processing are their peel, kernel and seed, accounting for 25–40% of the fruit’s mass (Banerjee et al., 2016), here referred to as Mango Processing Waste (MPW). The main groups of secondary metabolites found in mango fruits are flavonoids, xanthonoids, anthocyanins and carotenoids, with the xanthonoid mangiferin and the flavonoid hyperoside often appearing as the main bioactive components in mango pulps and peels (Berardini et al., 2005a; 2005b). Both substances have been studied in more intensity regarding their therapeutical properties, and mangiferin has been found to be an anti-cancer drug with low toxicity to the human body (Morozkina et al., 2021), as well as a powerful anti-inflammatory, among other activities related to pharmaceutical and cosmetic uses (Quadri et al., 2019). Hyperoside (quercetin-3-O-galactoside), commonly found in several plant species, has been tested for several pharmacological properties and is a potential drug candidate for different applications (Raza et al., 2017). Beyond pharmaceutical applications, natural products could be used as biopesticides representing a green alternative to synthetic pesticides (Wilson et al., 2013).

Regarding pesticidal or biocidal activity of mangiferin and hyperoside, fewer studies are found in the literature. Emam and colaborators tested the larvicidal activity of mango kernel extract and isolated mangiferin against the house mosquito *Culex pipiens L.* (Emam et al., 2021)*.* An ethanolic extract containing mangiferin was active against plant-pathogen fungi *Colletotrichum brevisporum* (Gómez-Maldonado et al., 2020). Crude extracts from different plants containing hyperoside were also tested for their herbicidal (Albouchi et al., 2013; Puig et al., 2018) and insecticidal activities (García-Bores et al., 2018).

The extraction and isolation of target compounds from complex matrices such as plants and microorganisms are costly processes, either economically or environmentally due to solvent and energy consumption (Chemat et al., 2020). The extraction of mangiferin and/or hyperoside from mango waste has been previously studied with ethanolic mixtures using the Homogenizer-Assisted Extraction (HAE) technique (Zuin et al., 2020), Microwave-Assisted Extraction (MAE) (Zou et al., 2013; Dorta et al., 2014; Segatto et al., 2021), Ultrasound-Assisted Extraction (UAE) (Kim et al., 2012; Safdar et al., 2017) and Matrix Solid-Phase Dispersion (MSPD) (Segatto et al., 2021). Solvent choice plays an important role for optimizing the extraction of bioactive compounds, as solubility, viscosity and other physical-chemical properties directly affect the extraction yield and are dependent of the chemical/molecular properties of the targets. Therefore, it is important to assess the effectiveness and sufficiency of each solvent alternative in terms of efficiency without compromising its impact in the product’s life cycle, and to find the best balance between these factors (Milescu et al., 2020; Zuin et al., 2021).

Among the traditionally used organic solvents, alcohols such as ethanol and methanol are some of the most commonly used solvents for the extraction of medium-high polarity phenolic compounds (Chaves et al., 2020). Produced in high scales from renewable sources and with a low price (Rossi et al., 2021), ethanol has a low toxicity and low persistency in nature, being classified as a one of the safest solvents in several solvent guides (Alder et al., 2016; Prat et al., 2016). Although the numerous advantages of using ethanol-water mixtures, the extraction of some secondary metabolites is hindered by the limited solubility of these mixtures (Liu and Chen, 2008; Ferreira and Pinho, 2012; Bogdanov, 2014). Alternative solvents for the extraction of plant constituents have been studied in recent years, featuring natural or naturally-derived solvents, or bio-solvents (e.g. Cyrene, 2-MeTHF, ethyl lactate), supercritical fluids, subcritical water, Deep Eutectic Solvents (DES), and Ionic Liquids (ILs) (Moity et al., 2014; Cvjetko Bubalo et al., 2018; Milescu et al., 2020).

ILs are solvents formed from a mixture of a pair of solid salts, organic or inorganic, that when combined have their melting point below 100°C, and are commonly liquid at room temperature (Han and Armstrong, 2007). Due to their non-volatile nature, in contrast to organic solvents, and good solubility parameters, ionic liquids were discussed primarily as green solvents for a myriad of applications (Rogers and Seddon, 2003). However, there are concerns on scaling up processes using ILs due to their environmental fate, including properties such as persistence and biodegradability, as most of “first generation” ionic liquids and their degradation products perform very poorly on toxicity assays and may be a danger to environmental and human systems if not properly treated (Haiß et al., 2016). Nevertheless, the customizability of ILs, as anions and cations can be interchanged to give different solubility and biodegradation parameters, is another important feature that helps finding optimum extraction yields for bioactive compounds from natural products through greener processes (Dai et al., 2013). In addition, newer biodegradable ILs have been discovered in recent years, which could surpass the disadvantages related to their environmental fate if these solvents are commercially available in the future at a reasonable price (Amsel et al., 2022).

1-alkyl-3-methylimidazolium cations ([C_n_MIm]) are one of the most available ILs found in the market and that they have been used frequently in studies regarding the extraction of secondary metabolites from plants (Bogdanov, 2014). The alkyl length (n) present in these cations, which usually variates between 2–10 carbons, controls the solvents’ polarity and therefore the ability to solubilize the desired substances. 1-butyl-1-methylpyrrolidinium bromide ([C_4_MPyrr] Br) was also has common appearances in previous studies and is largely available for purchase worldwide. Also widely available, choline acetate, 2-hydroxyethylammonion formate and 1-ethylpyridinium bromide are commonly linked to an enhanced biodegradability, although only the former can be considered readily degradable according to the closed bottle test (Jordan and Gathergood, 2015). On the other hand, ILs with imidazolium cations, including [C_8_MIm] Cl, are linked to high toxicity (Liu et al., 2015) and medium to low biodegradability (Romero et al., 2008). Prices are also not attractive, reaching up to 500 US Dollars for 500 g packages of [C_8_MIm] Cl (Alichem, 2022). As it has been often found that ILs show higher extraction yields in comparison to conventional solvents, it is important to understand in which degree this enhancement in performance takes place and if it is sufficient to overcome their poor biodegradability and high toxicity, depending on which IL was studied.

Regarding down-stream processing of extracts to purify the active compounds, the most common isolation (or separation) methodologies involve the use of a high volume of organic solvents, as in chromatographic techniques, which increases the cost and the environmental impact of the process, escalating accordingly to the desired purity of the isolated compound (Zhang et al., 2018). The use of crude extracts as pesticidal agents is desirable, therefore, if sufficient biological activity can be achieved, considering also other properties that should be taken into account when formulating a product, such as stability and selectivity. In fact, synergy is expected when using crude extracts, as the interaction of different molecules could combine to a higher level of mortality of the target organism, as well as other positive effects such as enhanced target absorption rates, protection against degradation and resistance inhibition (Gilbert and Alves, 2003; Rasoanaivo et al., 2011). However, when using an extract for pesticidal applications, the extract solvent will enter the environment and therefore needs to have a low ecotoxicity and to be non-persistent.

This study aims at a more holistic approach towards green and more sustainable extraction of bioactive compounds from mango processing waste. Therefore, commonly used ILs such as 1-alkyl-3-methyl-imidazolium cations were compared to greener ILs (e.g., choline acetate and 2-hydroxyethylammonion formate). HAE has been used as a quick, efficient and inexpensive extraction technique. In an initial screening approach, the most promising imidazolium IL and the most promising greener IL in terms of efficiency and biodegradability were selected. For these ILs the extraction was optimized using a Box-Behnken experimental design with the aid of Response Surface Methodology (RSM). The optimum extraction recoveries were compared to those obtained with 70% ethanol, an optimized mixture obtained from a previous study with HAE (Zuin et al., 2020). Furthermore, the algicidal activity of the extracts obtained with non-persistent solvents (choline acetate + ethanol) was investigated. To assess the activity of the extract in comparison to the single substance, mangiferin, hyperoside and a 1:1 mixture of both substances was tested as well.

## 2 Materials and methods

### 2.1 Chemicals and reagents

Mangiferin standard was purchased from Sigma-Aldrich (assay ≥98.0%) and Hyperoside from the HWI group (prime reference standard). All ionic liquids were purchased from Iolitec (Heilbronn, Germany) with 97% or higher purity, as assessed by the company. Standard stock solutions of 200 mg L^−1^ of mangiferin and hyperoside, were prepared by accurately weighing the proper mass of each and adding them to a 1 M [C_4_MIm] Br solution.

### 2.2 Mango processing waste sample

Organic mango processing waste (*Mangifera indica L.,* var. Palmer) was obtained from a commercial processing unit in Itirapina (São Paulo, Brazil) as a mixture of mango peels, puree and seeds. Samples were refrigerated on site and transported to the laboratory, where they were stocked in - 20°C until being properly processed. Frozen samples were dried in a convection oven until constant mass, homogenized, blended and sieved, obtaining a powder with particle size <500 μm, which was stored protected from light and humidity until extraction.

### 2.3 Liquid chromatography

Liquid chromatography was performed using a HPLC (High-Performance Liquid Chromatography) system Shimadzu coupled with UV-vis and Photodiode Array UV (PDA) detectors, which allowed the detection of the analytes in the UV-Vis range and the observation of the UV-Vis profile. The separation was achieved with Nucleodur C8 column (Macherey-Nagel, 3 μm; 2 × 125 mm), using water with formic acid (0.1%) and methanol as mobile phase at 0.3 ml min^−1^, injection volume of 2 μL, column temperature of 35°C and the selected wavelength of 350 nm for both analytes. The gradient started with the percentage of methanol in 10% and raised through the chromatographic run as follows: 0 min, 10%; 4 min, 20%; 14 min, 30%; 21 min, 40%; 24 min, 100%; 29 min, 100%; 30 min, 10%; 40 min, 10%.

### 2.4 Calibration curve

A calibration curve was performed in order to predict the concentration of the analytes based on their peak areas obtained through the liquid chromatography methodology. The standard solution was appropriately diluted to the following concentrations of mangiferin (in duplicate) and hyperoside (in quadruplicate): 150, 100, 75, 50, 25, 10, 5 and 1 mg L^−1^. The correlation between these concentrations and the means of their related peak areas resulted in the calibration curves. The slope, intercept and the correlation coefficient (*R* ^2^) were calculated according to the least squared method and are shown in Table 1. The limits of detection (LOD) and of quantification (LOQ) can also be seen in the same table, as calculated accordingly to the ICH guidelines (ICH, 2005).

**TABLE 1:** Calibration curves of mangiferin and hyperoside with HPLC-PDA.

Compound	Equation	*R* ^2^	LOD	LOQ
			mg L^−1^	mg L^−1^
**Mangiferin**	y = 7970.67x - 4919.90	0.9998	2.47	8.24
**Hyperoside**	y = 15208.46x - 4519.21	0.9998	2.87	9.56

### 2.5 HAE extraction process

For the Homogenizer-Assisted Extraction (HAE)—a technique that demonstrated to be effective considering both analytical and green and sustainable criteria for the extraction of bioactive compounds from fruit waste (Zuin and Ramin, 2018; da Silva Francischini et al., 2020)—samples were accurately weighed accordingly to the sample/solvent ratio of each run and transferred to a 15 ml plastic tube containing 5 ml of the solvent. Extraction was performed at room temperature using IKA’s T25 Basic ULTRA-TURRAX^®^ at 16,000 rpm. For the screening and activity tests, the extraction was performed for 10 min with a 10% sample/solvent ratio, while the tests for optimization of the selected ILs, both time and sample/solvent ratio vary accordingly to the experimental design. All mixtures were then centrifuged at 4,000 rpm for 10 min (Hettich Rotanta 460); the supernatants were collected and filtered using a 0.45 µm PTFE filter (Chromafil^®^ Xtra PES-45/25) and the resulting extracts were further analysed by liquid chromatography. The results are expressed as the concentration in mg kg^−1^ of MPW (dry weight). For comparison purposes, optimum parameters obtained previously for ethanol-water mixtures as solvents with HAE (Zuin et al., 2020) were used to prepare an ethanolic extract, with 70% ethanol in water as a solvent and the same extraction conditions used for the screening experiments (10 min and 10% sample/solvent ratio).

### 2.6 Screening of ILs

Commercially available ionic liquids were selected according to their use in past works for the extraction of secondary metabolites from plant matrices, their biodegradability according to the literature and their availability to be purchased. Ethyl (C_2_), butyl (C_4_), hexyl (C_6_), octyl (C_8_) and decyl (C_10_) variants of 1-alkyl-3-methylimidazolium ([C_n_MIm]) were tested, with bromine as their anion pair, as well as 1-octyl-3-methylimidazolium chloride, the same C_8_ variant, but with chloride as the anion. The other selected ILs were: 1-butyl-1-methylpyrrolidinium bromide ([C_4_MPyrr] Br), choline acetate, 2-hydroxyethylammonium formate and 1-ethylpyridinium bromide. The extractions were performed using aqueous solutions of 1 M of each ionic liquid in triplicate, following the abovementioned procedure.

### 2.7 Optimization of extraction with selected ILs

#### 2.7.1 Experimental design

A Box-Behnken design with three variables and a triplicate on the central point, resulting in 15 experiments, was used to optimize the extraction parameters for the ILs selected from the screening process according to their extraction performance and biodegradability. The variables selected were: concentration of the IL in the aqueous solution (X_1_), time (X_2_) and sample-to-solvent ratio (X_3_). Their minimum and maximum levels, as well as central points are expressed in Table 2. The parameter levels of each experiment that constitute the Box-Behnken design will be presented in the results section, along with the responses.

**TABLE 2:** Parameters used in Box-Behnken design for the optimization of MPW extraction.

Symbol	Real variables	Coded variables		
		Min (-1)	CP (0)	Max. (+1)
X_1_	IL concentration (M)	0.2	1.1	2
X_2_	Time (min)	5	17.5	30
X_3_	Sample/Solvent ratio (g ml^−1^)	0.05	0.1	0.15

CP = central point.

#### 2.7.2 Response surface methodology

In order to assess the influences of each variable and find optimum parameters, a Response Surface Methodology (RSM) was employed using the responses obtained in the experiments determined by the Box-Behnken design. A second-order (quadratic) polynomial model was calculated for each analyte obtained through each selected IL. Equation 1 shows the polynomial model in which the responses were fit, obtaining the coefficients (β), which allowed us to determine the equation that rules the responses (y), as affected by the variables (X_1_-X_3_).

$$y=\beta_{0}+\overset{k}{\underset{j=1}{\sum }}\beta_{j}X_{j}+\overset{k}{\underset{j=1}{\sum }}\beta_{jj}X_{j}^{2}+\sum \overset{k}{\underset{i<j=2}{\sum }}\beta_{ij}X_{i}X_{j}$$ (1)

The polynomial regression was obtained using GNU Octave (version 4.2.1). The surface and contour plots were plotted using the software OriginPro 9.0 and the optimum variables were calculated using Microsoft Excel (Professional Plus 2016) and Solver add-in (evolutionary methodology).

### 2.8 Algicidal activity

Algae growth inhibition was investigated for the choline acetate and ethanol extract, as well as for mangiferin, hyperoside and a 1:1 mixture of both substances, according to the OECD guideline 201 and in subject to its validation criteria. *Raphidocelis subcapitata* (Culture Collection of Algae at Goettingen University, Germany, SAG 61.81, formerly known as *Pseudokirchneriella subcapitata*) was chosen as test organism and cultured in OECD medium (Supplementary Table S1). The assay was carried out in 24 well plates (Greiner Bio-One, Germany) with a start cell density of 10,000 cells mL^−1^ and a total volume of 2 ml. Different volumes of test substances (DMSO stock solution) or extracts were added to obtain six to nine different concentrations. Additional DMSO or extraction solvent was added to reach an equal amount of organic solvent in each well. Growth controls and blanks were treated with the same volume of solvent. Each treatment was measured in duplicates. The ethanolic extracts was sterilized by filtration before application (syringe filter RC 0.2 µm, Machery-Nagel). Algae and test substance or extracts were incubated for 72 h in an incubator (AlgaeTron AG 130-ECO, Photo Systems Instruments) at 23 ± 2°C under continuous illumination (100 μE m^2^ s^−1^) and agitation at 160 rpm using a shaker (Unimax 1,010, Heidolph Instruments). Cell density was measured at 24-h intervals via chlorophyll a fluorescence (excitation: 450 nm, bandwidth 40 nm/emission 680 nm, bandwidth 30 nm) using a plate reader (Synergy HT, BioTek Instruments). To cover the exponential growth, the fluorescence calibration ranged from 5 × 10^3^ to 1.5 × 10^6^ cells mL^−1^ (Supplementary Figure S1). For a higher precision at low cell densities, these were determined using calibration points 5 000 to 100,000 cells mL^−1^. Using this range, limits of detection (2 051 cells mL^−1^) and quantification (7 712 cells mL^−1^) were calculated with DINTEST 2000 according to DIN 32645 (result uncertainty 33.3%, probability of error 5%). Before measurements, plates were sealed with parafilm in addition to the plate’s lid and inverted 10 times to ensure homogenous suspension of algae cells. Each assay was repeated once yielding 4 replicates of each treatment in total. Growth inhibition ( ${\%I}_{µ})$ based on the growth rate (µ) was calculated according to Equations 2, 3. Start cell density was set to 10,000 cells mL^−1^. Dose-response curves (%inhibition vs. log of concentration) were plotted and EC_50_-values obtained by linear regression of selected data point. Each replicate was fitted individually and then an average EC_50_-values was calculated.

$$µ=\frac{\ln{cell\;density}_{72h}-{\ln cell\;density}_{0}}{\Delta t}$$ (2)

$${\%I}_{µ}=\frac{{µ}_{control}-{µ}_{sample}}{{µ}_{control}}*100$$ (3)

## 3 Results and discussion

### 3.1 Screening of Ionic liquids

**FIGURE 1:**
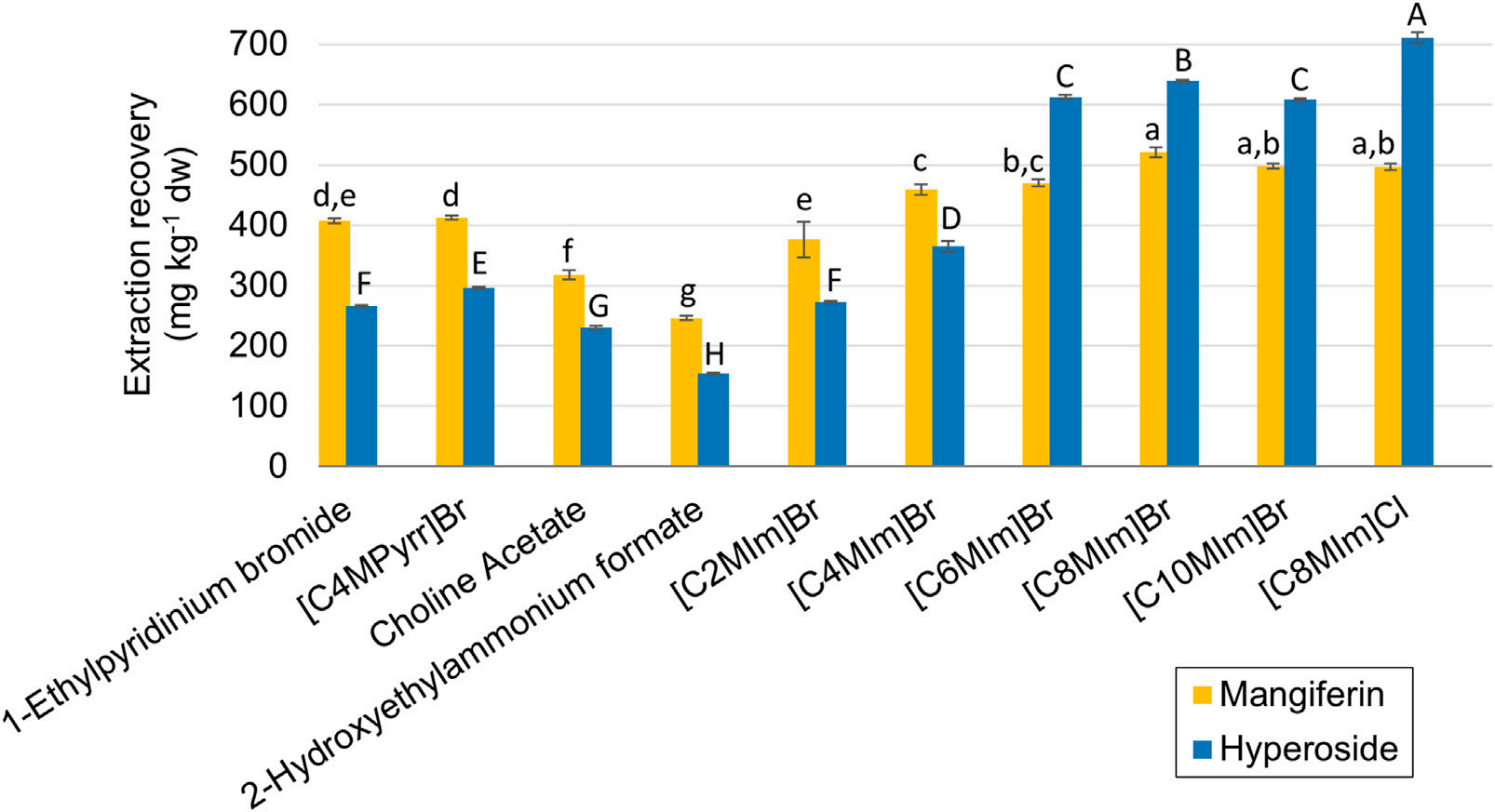
Extraction recoveries of mangiferin and hyperoside for the screening experiments. Error bars are expressed in terms of the standard deviation from the triplicates for each experiment. The letters represent the statistical differences between the averages according to the Tukey test (*p* < 0.05) for mangiferin (lowercase) and hyperoside (uppercase).

As stated before, ten different ILs were selected for the screening step based on the most used compounds for natural products extraction and those ILs that are considered less persistent and show better biodegradability aspects. Figure 1 shows the results for the extraction recoveries of mangiferin and hyperoside from mango processing waste for this screening procedure. Imidazolium-based ILs showed higher extraction recoveries for both analytes, which peaked at the 1-octyl-imidazolium cation, when compared to the other alkyl chain lengths. Comparison between two different anions paired to [C_8_MIm] showed no statistical difference between the results for mangiferin (497.0 and 520.8 mg kg^−1^ for chlorine and bromine, respectively) but a superior yield of hyperoside extraction for the chlorine anion, 710.2 mg kg^−1^, against 638.5 mg kg^−1^ from bromine. Therefore [C_8_MIm] Cl was selected for further optimization. The other ILs showed limited extraction efficiency, with no higher than 412.2 mg kg^−1^ of extraction recovery for mangiferin and 296.7 mg kg^−1^ for hyperoside ([C_4_MPyrr] Br). Although not presenting a similar response to the other tested ILs, the extraction with choline acetate was further investigated in the optimization step due to its biodegradable nature.

### 3.2 Optimization of extraction parameters for [C_8_MIm] Cl and choline acetate

The Box-Behnken experimental design prepared for the optimization of the extraction recoveries of mangiferin and hyperoside using aqueous solutions of [C_8_MIm] Cl and choline acetate can be seen in Table 3, as well as the responses obtained in each experiment. An initial observation of the experiments and responses shows that variable X_1_ (concentration of the IL) has a significant positive effect on the extraction recovery of both analytes, as maximum responses were obtained when this variable was in its maximum level (experiments 6, 7, 10 and 11). Little difference between the pairs of experiments 6/7 and 10/11 shows that variables X_2_ (time) and X_3_ (sample/solvent ratio) have small effect on the final result when compared to X_1_, as it will be further analyzed by RSM. Although higher values of response were obtained when comparing with the screening experiments, the extraction recoveries obtained using choline acetate remained smaller than using [C_8_MIm] Cl.

**TABLE 3:** Box-Behnken experimental design and extraction recovery responses for the optimization of HAE with [C_8_MIm] Cl and choline acetate ionic liquids.

	Variables						Responses			
	IL concent. (X_1_)		Time (X_2_)		Sample/solvent (X_3_)		[C_8_MIm] Cl		Choline acetate	
	(M)		min		g mL^−1^		mg kg^−1^		mg kg^−1^	
Exp	Cod	Real	Cod	Real	Cod	Real	Mangiferin	Hyperoside	Mangiferin	Hyperoside
1	0	1.1	0	17.5	0	0.10	503.7	737.9	300.5	154.8
2	0	1.1	0	17.5	0	0.10	511.8	734.4	297.8	152.1
3	0	1.1	0	17.5	0	0.10	513.7	737.8	297.6	152.2
4	−1	0.2	−1	5.0	0	0.10	279.2	115.3	174.7	29.2
5	−1	0.2	1	30.0	0	0.10	299.0	132.0	153.7	31.7
6	1	2	−1	5.0	0	0.10	726.9	811.2	414.8	303.6
7	1	2	1	30.0	0	0.10	732.6	882.4	415.8	352.7
8	−1	0.2	0	17.5	-1	0.05	340.0	170.8	189.9	18.4
9	−1	0.2	0	17.5	1	0.15	237.4	86.6	141.6	29.0
10	1	2	0	17.5	−1	0.05	632.1	835.9	378.1	383.8
11	1	2	0	17.5	1	0.15	787.7	915.5	422.9	346.6
12	0	1.1	−1	5.0	−1	0.05	433.1	656.4	371.8	202.3
13	0	1.1	−1	5.0	1	0.15	527.8	696.3	306.3	150.3
14	0	1.1	1	30.0	−1	0.05	524.9	695.3	335.4	164.0
15	0	1.1	1	30.0	1	0.15	527.4	763.8	316.5	159.5

A quadratic polynomial model was calculated for each IL and analyte. The equation coefficients and the ANOVA table for each model can be found in the Supplementary Material (Supplementary Table S2, S3). ANOVA shows that a statistically significant (*p* ≤ 0.05) regression model was obtained for all equations. Except for the model obtained for mangiferin using [C8MIm] Cl, the lack of fit was considered significant when compared to the pure error calculated from the triplicates. Besides that, regression coefficients (*R* ^2^) were considered adequate (>0.99 for all models) and the calculated models were plotted into response surfaces. At the best experiment’s condition (experiment 11, Table 3), the differences between the experimental (787.70 and 915.53 mg kg^−1^) and the calculated (798.08 and 923.68 mg kg^−1^) results are small, at around 1% difference, showing that a sufficient model has been achieved for the optimized region (mangiferin and hyperoside, respectively).

**FIGURE 2:**
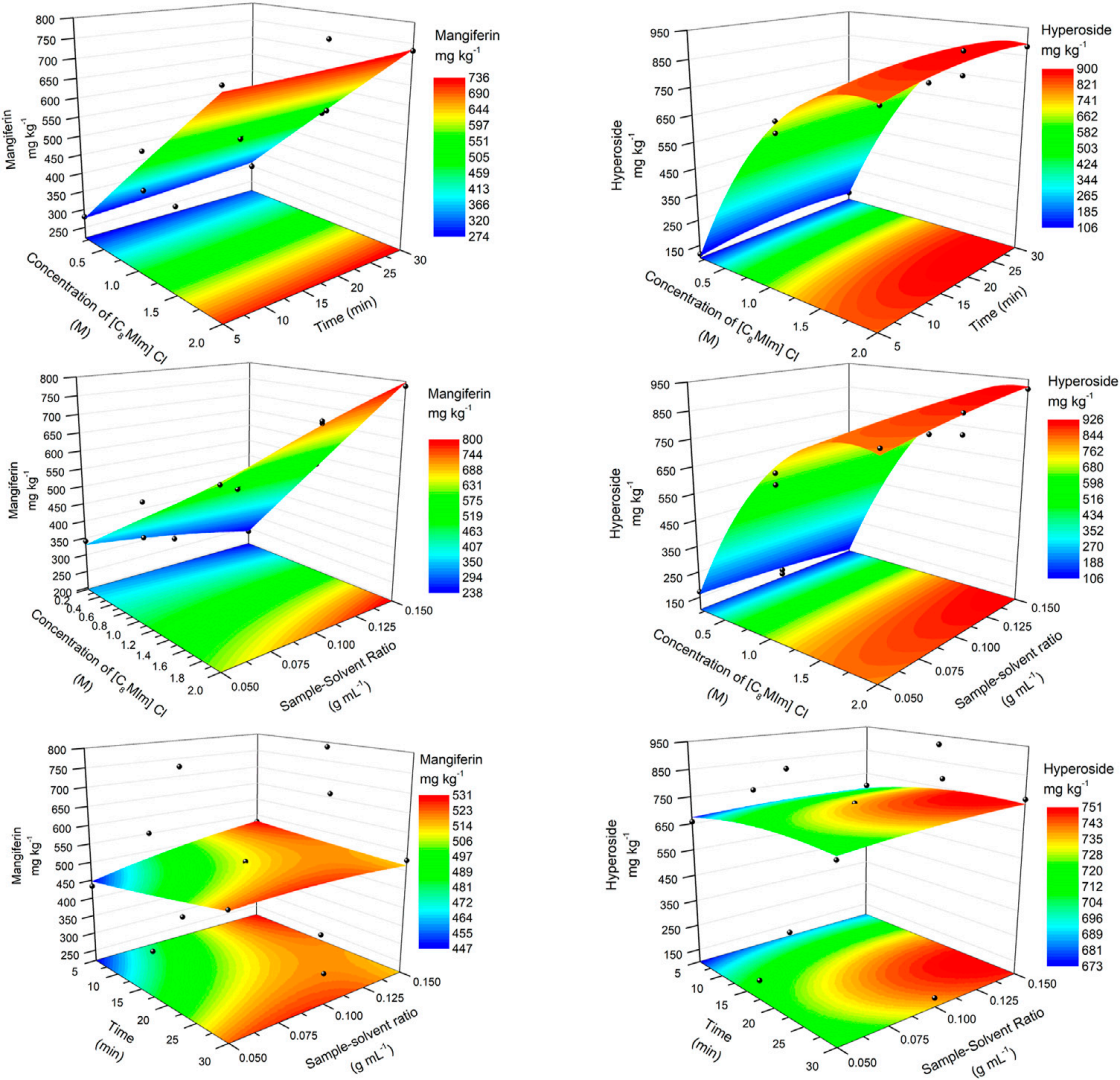
RSM plots for the optimization of the extraction of mangiferin (left) and hyperoside (right) using [C_8_MIm] Cl ionic liquid.

Figure 2 shows the response surface methodology for the extraction of both analytes using aqueous solutions of [C_8_MIm] Cl. The first two rows of graphs show the clear influence of the concentration of the IL on the response of mangiferin and hyperoside, and maximum responses obtained were in the region of 1.9–2.0 M. The horizontal format of the model in the time axis (first row) shows that this variable has a small effect on the response, although slightly higher concentrations were found at 20–30 min for hyperoside. In the second row, it is possible to observe that a higher response can be obtained at the maximum level of the sample/solvent ratio variable (0.15 g ml^−1^), for either mangiferin and hyperoside. The third row shows the smaller effect of both variables X_2_ and X_3_ due to the “flat” format of the surface. A calculated optimization of the variables based on the quadratic polynomial equation was made, obtaining: X_1_ = 2 M, X_2_ = 5.0 min, X_3_ = 0.15 g ml^−1^ for mangiferin (811.9 mg kg^−1^); X_1_ = 1.9 M, X_2_ = 28.4 min, X_3_ = 0.15 g ml^−1^ for hyperoside (944.79 mg kg^−1^).

**FIGURE 3:**
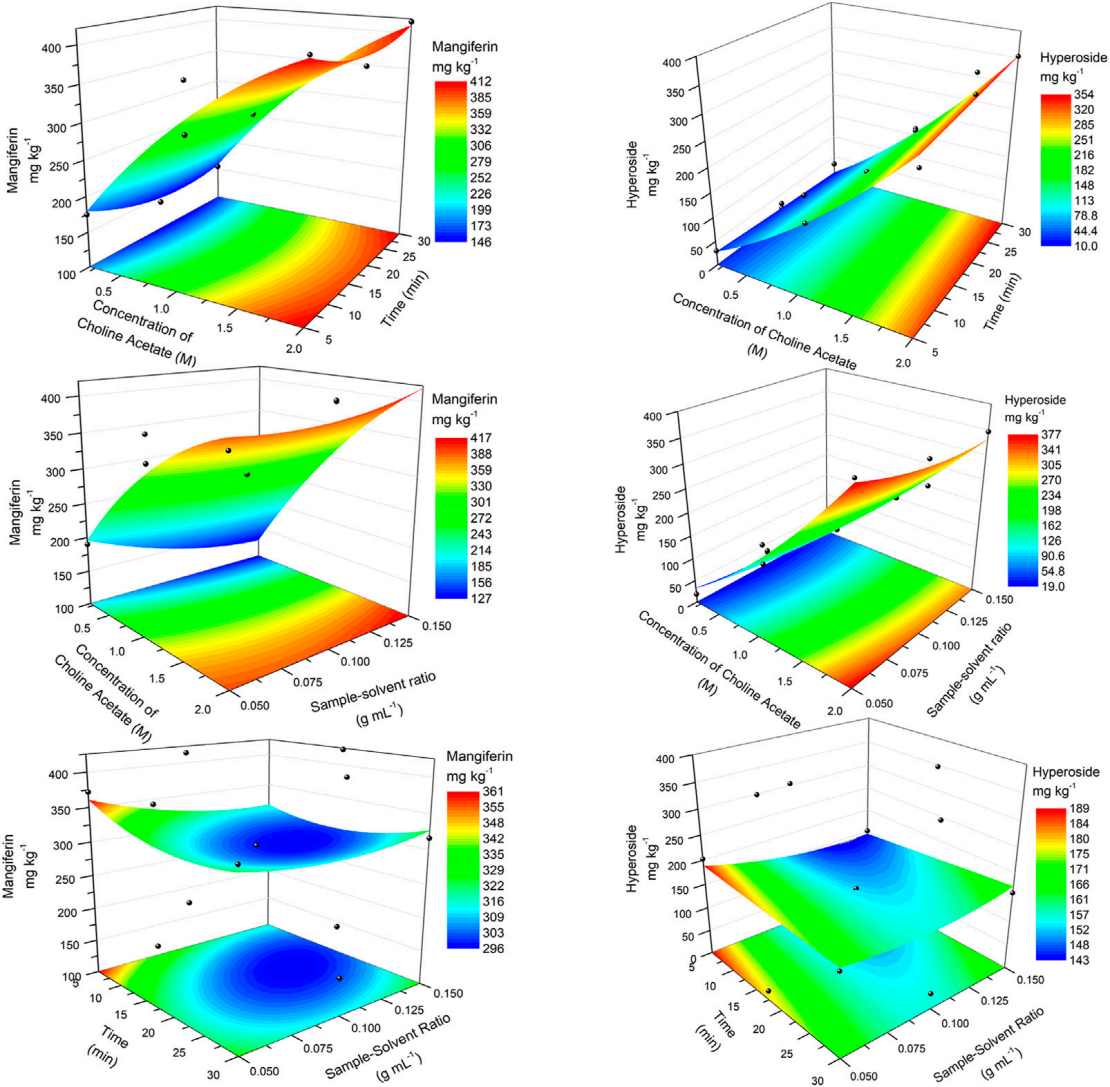
RSM plots for the optimization of the extraction of mangiferin (left) and hyperoside (right) using choline acetate ionic liquid.

Similar surfaces were obtained for the extraction with choline acetate (Figure 3). Again, the concentration of the ionic liquid was crucial for obtaining higher responses, which had maximum response at 2 M (first two rows). Comparing the best experimental results (experiments 11 and 10, Table 3) with the calculated results using the quadratic polynomial model for the same conditions (416.91 and 376.59 mg kg^−1^ for mangiferin and hyperoside, respectively), a difference of less than 2% was found. This shows a good approximation of the model at the optimum region of response. Flat surfaces on the third row of Figure 3 showed once again the smaller effects of the variations of parameters X_2_ and X_3_. Calculated maximum responses were found for mangiferin at 448.5 mg kg^−1^ (X_1_ = 2.0 M, X_2_ = 30.0 min, X_3_ = 0.15 g ml^−1^) and for hyperoside at 379.6 mg kg^−1^ (X_1_ = 2.0 M, X_2_ = 30.0 min, X_3_ = 0.05 g ml^−1^). As minimum effect was observed for the sample/solvent ratio, the highest tested level (0.15 g ml^−1^) can be assumed, since less solvent is used to obtain close to maximum extraction recoveries. On the other hand, no validation of the optimum parameters for both choline acetate and [C_8_MIm] Cl was performed and further comparison was made using the best experimental conditions obtained with the Box-Behnken design.

**FIGURE 4:**
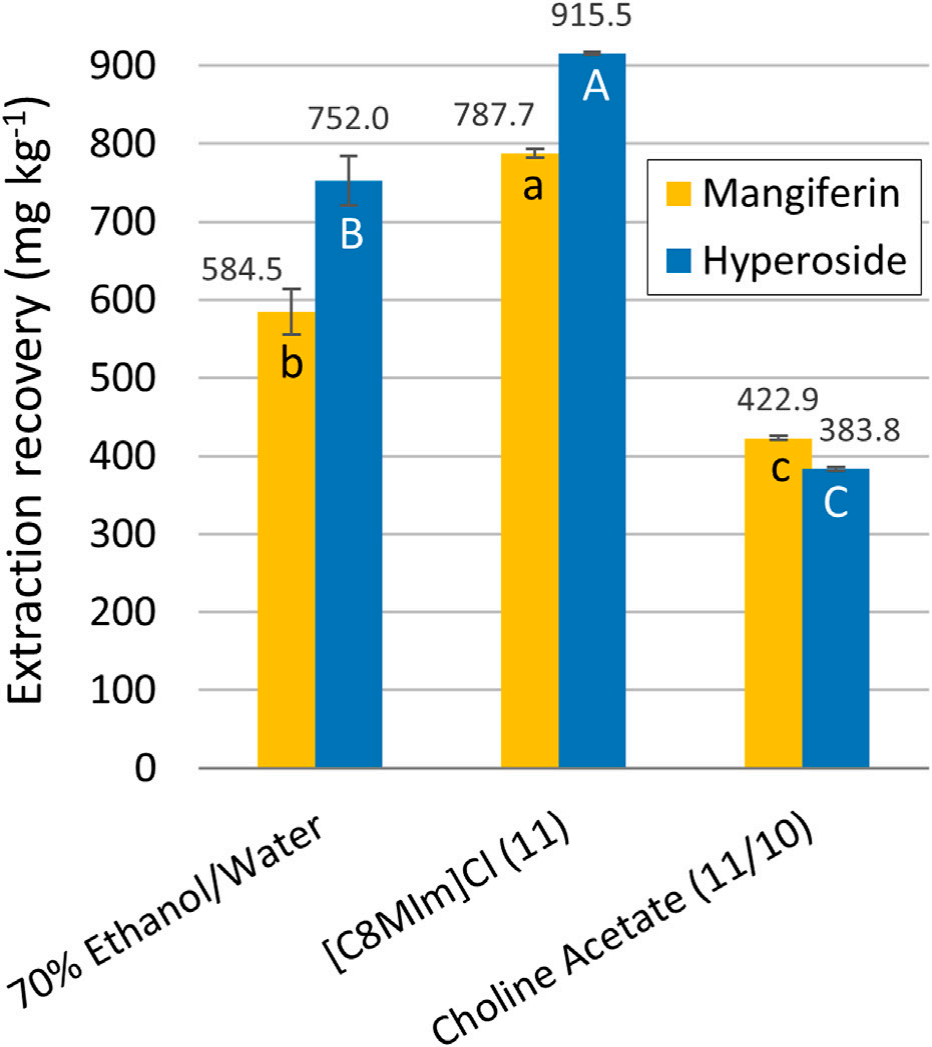
Comparison between the highest experimental extraction recoveries obtained with ethanol and ionic liquids aqueous solvents. The letters represent the statistical differences between the averages according to the Tukey test (*p* < 0.05) for mangiferin (lowercase) and hyperoside (uppercase).

For comparison purposes, an extraction experiment with ethanol/water as solvent was carried out using the parameters obtained in a previous optimization study (Zuin et al., 2020). In comparison with the best conditions found experimentally for each IL solvent [C_8_MIm] Cl achieved the best performance (Figure 4). A considerably higher (in average, 22%) yield was obtained for the non-biodegradable imidazolium IL (experiment 11, Table 3) when compared to the ethanolic counterpart. The ethanol-water mixture showed better results than the optimum biodegradable choline acetate-water mixture, with an average of 45% higher response (choline acetate extraction experiments 11 for mangiferin and 10 for hyperoside, Table 3). It is important to notice that, although having a lower extraction recovery, the results for the ethanolic extract are in the same order of magnitude of [C_8_MIm] Cl, which could be sufficient for justifying its use in this process, especially considering economic and environmental factors. In fact, ethanol is already inserted in a bioeconomy approach when produced from natural resources, even more when manufactured with second generation processes from agro-industrial waste, reducing the volume of residues generated in agricultural practices and avoiding the introduction of another land use competitor with food resources. Besides that, as already stated, the environmental fate of ethanol/water mixtures is considerably milder compared to [C_8_MIm] Cl, and its higher extraction efficiency makes it preferable than the extraction with choline acetate.

### 3.3 Algicidal activity

After extraction optimization, the two extracts obtained with the biodegradable solvents ethanol and choline acetate were investigated for their algae growth inhibition activity to explore their potential as e.g., antifouling agents. Due to the entry of pesticidal formulation into the environment, non-biodegradable solvents like [C_8_MIm] Cl are unsuited for these applications. However, the investigation of the extract obtained with choline acetate was hindered because it was unstable during storage at -20 °C and composition continued to change after unfreezing (Supplementary Figure S2). The changing composition of the choline acetate extract demonstrates the need to include stability considerations of the obtained extract into the choice of extraction solvent.

**FIGURE 5:**
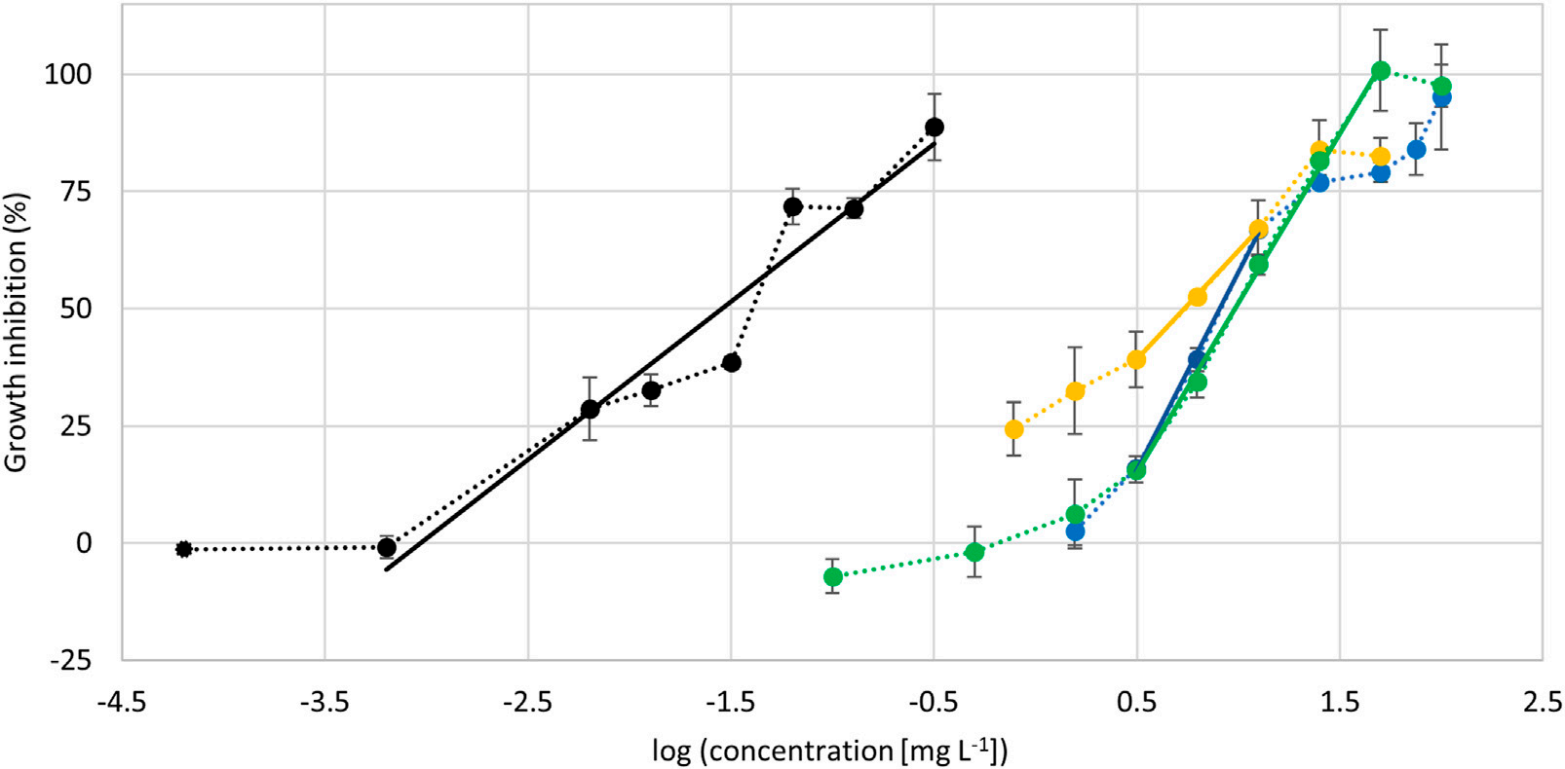
Dose-response curves showing the algae growth inhibition in dependency of test substance concentration. Black: treatment with ethanolic extract, concentration corresponds to the additive concentration of mangiferin and hyperoside contained in the extract. Yellow: treatment with mangiferin. Blue: treatment with hyperoside. Green: treatment with 1:1 mixture (w:w) of mangiferin and hyperoside. n = 4. Lines represent linear regressions used to determine EC_50_-values. Dotted lines represent a visual guide.

The ethanolic extract was suited for further investigation. It was stable during storage and sterile filtration did not affect the concentration of mangiferin or hyperoside (Supplementary Figure S3). Hence, the algae growth inhibition of the ethanolic extract, the two major compounds mangiferin and hyperoside, and a 1:1 mixture of both compounds were investigated using the freshwater microalgae *Raphidocelis subcapitata*. Dose-response curves were acquired and the EC_50_-values obtained (Figure 5). Mangiferin and hyperoside have similar EC_50_-values in the range of 5.5–13 mg L^−1^ showing a moderate effect on the algae growth (Table 4). Likewise, the mixture of these compounds results in a comparable effect with an EC_50_-value of 9.7 mg L^−1^ suggesting no synergetic effect of mangiferin and hyperoside. In contrast, the ethanolic extract strongly inhibits the algae growth. The EC_50_-value based on the additive concentration of mangiferin and hyperoside contained in the extract was found to be two orders of magnitude lower with 0.015 mg L^−1^. The highest concentration (0.32 mg L^−1^) shown in the dose response curve resulted in a growth inhibition of 89 ± 7%. The addition of a larger volume of extract (10 µL ≙ 0.64 mg L^−1^ final concentration in well) resulted in a cell density below the quantification limit of 7 712 cells mL^−1^. Since the start cell density was 10,000 cells mL^−1^, treatments with 10 µL of ethanolic extract resemble a growth inhibition >100%. The high activity of the extract may be caused by additional ingredients or synergistic effects of unknown ingredients with mangiferin or hyperoside. Synergistic effects were also proposed for different plant extracts investigated for pesticidal activities (Céspedes et al., 2014; Gillmeister et al., 2019).

**TABLE 4:** EC_50_-values of tested samples given in mg L^−1^ and μmol L^−1^. For the mixture and the ethanolic extract concentrations refer to additive concentration of both substances.

Sample	EC_50_ (mg L^−1^)	EC_50_ (µmol L^−1^)
Mangiferin	5.5 ± 1.0	13 ± 2.4
Hyperoside	13 ± 5	28 ± 11
Mangiferin-hyperoside 1:1 (w:w)	9.7 ± 0.7	22 ± 2
70% Ethanol extract	0.015 ± 0.001	0.033 ± 0.002

To date, only few studies have investigated the pesticidal activities of mango waste extracts (Gómez-Maldonado et al., 2020; Emam et al., 2021). To our knowledge, we are the first to test its algae growth inhibition and present a direct comparison of the extract’s activity to two of the major components. Growth inhibition of different algae species and cyanobacteria was previously observed for structurally closely related flavonoids like quercetin, rutin and quercetin-3-β-D-glucose, (D’Abrosca et al., 2006; Jiang et al., 2012; Xiao et al., 2019). Additionally, plant extracts were investigated for algicidal activity including one obtained from pomegranate peel (Xiao et al., 2014; Chen et al., 2019; Zhu et al., 2019). However, this is often performed to find most active fractions and subsequently an active ingredient. In regard of the results obtained in this study, the utilization of extracts may be more effective for pesticidal applications. The algicidal activity of the mango waste ethanolic extract observed in this study could lead to its utilization as algicide in antifouling and other applications.

## 4 Conclusion

This study set out to evaluate sustainability aspects of the solvent choice for extraction of high value compounds from agro-industrial waste taking into account their sources, performance, biodegradability and furthermore their suitability for possible applications of the extract. For the latter, we investigated the biocidal activity of the obtained extracts against algae.

Although [C_8_Mim] Cl showed higher extraction recoveries, due to its production from non-renewable resources, high toxicity and non-biodegradability, the advantages in comparison to ethanol, which showed only slightly lower responses, are not evident. The ethanol extract further outcompetes the extraction with choline acetate in yield and stability of the extract’s composition. It is important to notice that temperature was not a parameter assessed at this study, and results may have been different at higher temperatures. On the other hand, using room temperature is advised and should be encouraged in the development of sustainable processes, keeping in mind the balance between sustainability and efficiency (sufficiency).

Aiming at using the crude extract and exploiting its potential synergistic activity instead of isolated compounds and thereby bypassing energy- and resource-intensive isolation steps, the choice of solvent has to be fitted to the application as well. Using non-biodegradable ILs to obtain pesticidal active extract which are then used in agriculture and other external applications could cause severe environmental pollution. In this study, a higher growth inhibition of the ethanol extract in comparison to the single compounds and a 1:1 mixture was confirmed. Therefore, ethanol can be viewed as a cheaper, greener alternative—in comparison to the tested ILs—for the extraction of some of the main bioactive compounds from mango processing residues, as well as effectively being used directly as a formulation solvent to be applied for algicidal activity. This also means to design and adopt greener and more sustainable analytical approaches that can be transferred to larger and commercial scales, taking into account simplicity, sufficiency and functionality. As the scientific interest on these bioactive natural products grow, formulation, stability and economic viability studies should follow. Those are crucial steps towards finding green and sustainable alternatives of biocides and pesticides, one of the most important challenges of human development for the near future.

## Data Availability

The original contributions presented in the study are included in the article/Supplementary Material, further inquiries can be directed to the corresponding author.
