# Supplementary material for: Ionic liquids vs. ethanol as extraction media of algicidal compounds from mango processing waste
Source: Front Chem. 2022 Sep 16;10:986987. doi: 10.3389/fchem.2022.986987 (PMC9523220; doi:10.3389/fchem.2022.986987)
Supplement: Supplementary file 1 [file DataSheet1.docx]

| Substance | Concentration (g L^-1^) |
| --- | --- |
| NH_4_Cl | 1.5 |
| MgCl_2_ * 6H_2_O | 1.2 |
| CaCl_2_ * 2H_2_O | 1.8 |
| MgSO_4_ * 7H_2_O | 1.5 |
| KH_2_PO_4_ | 0.16 |
| FeCl_3_ * 6H_2_O | 0.064 |
| NaEDTA * 2H_2_O | 0.1 |
| H_3_BO_3_^a^ | 0.185 |
| MnCl * 4H_2_O | 0.415 |
| ZnCl_2_ | 0.003 |
| CoCl_2_ * 6H_2_O | 0.0015 |
| CuCl_2_ * 2H_2_O | 0.00001 |
| Na_2_MoO_4_ * 2H_2_O | 0.007 |
| NaHCO_3_ | 50 |

**Supplementary Table 1.** Composition of the OECD medium used in the algicidal assay.

| Source | Sum of squares | | Degrees of freedom | | Mean of square | *F* value | *p* value |  |
| --- | --- | --- | --- | --- | --- | --- | --- | --- |
|  |  |  |  |  |  |  |  |  |
| **Mangiferin (mg kg^-1^)** | | |  | |  |  |  |  |
| Model | 395070 | | 9 | | 43897.0 | 212.88 | 6.40 x 10^-6^ |  |
| Linear | 375949 | | 3 | | 125316 | 607.7 | <0.001 |  |
| X_1_ | 371421 | | 1 | | 371421 | 1801.15 | <0.001 |  |
| X_2_ | 1707 | | 1 | | 1707 | 8.28 | 0.035 |  |
| X_3_ | 2821 | | 1 | | 2821 | 13.68 | 0.014 |  |
| Square | 286 | | 3 | | 95 | 0.46 | 0.721 |  |
| X_1_*X_1_ | 17 | | 1 | | 17 | 0.08 | 0.785 |  |
| X_2_*X_2_ | 13 | | 1 | | 13 | 0.06 | 0.814 |  |
| X_3_*X_3_ | 253 | | 1 | | 253 | 1.23 | 0.319 |  |
| 2-Way Interaction | 18836 | | 3 | | 6279 | 30.45 | 0.001 |  |
| X_1_*X_2_ | 49 | | 1 | | 49 | 0.24 | 0.646 |  |
| X_1_*X_3_ | 16658 | | 1 | | 16658 | 80.78 | <0.001 |  |
| X_2_*X_3_ | 2128 | | 1 | | 2128 | 10.32 | 0.024 |  |
| Residual | 1031.0 | | 5 | | 206.2 | 4.77 |  |  |
| Total | 396100 | | 14 | | 28293.0 |  |  |  |
| Pure error | 56.36 | | 2 | | 28.2 | 11.53 | 0.081 |  |
| Lack of fit | 974.7 | | 3 | | 324.9 | 19.16 |  |  |
| R² | 0.997 | | 0.999 | |  |  |  |  |
| R² (máx) | 1.000 | | 1.000 | |  |  |  |  |
| **Model Equation** | $\boldsymbol{Y}\boldsymbol{=}\boldsymbol{509}\boldsymbol{.}\boldsymbol{72}\boldsymbol{+}\boldsymbol{215}\boldsymbol{.}\boldsymbol{47} \boldsymbol{X}_{\boldsymbol{1}}\boldsymbol{+}\boldsymbol{14}\boldsymbol{.}\boldsymbol{61} \boldsymbol{X}_{\boldsymbol{2}}\boldsymbol{+}\boldsymbol{18}\boldsymbol{.}\boldsymbol{78} \boldsymbol{X}_{\boldsymbol{3}}\boldsymbol{-}\boldsymbol{2}\boldsymbol{.}\boldsymbol{15} {\boldsymbol{X}_{\boldsymbol{1}}}^{\boldsymbol{2}}\boldsymbol{+}\boldsymbol{1}\boldsymbol{.}\boldsymbol{85} {\boldsymbol{X}_{\boldsymbol{2}}}^{\boldsymbol{2}}\boldsymbol{-}\boldsymbol{8}\boldsymbol{.}\boldsymbol{27} {\boldsymbol{X}_{\boldsymbol{3}}}^{\boldsymbol{2}}\boldsymbol{-}\boldsymbol{3}\boldsymbol{.}\boldsymbol{50} \boldsymbol{X}_{\boldsymbol{1}} \boldsymbol{X}_{\boldsymbol{2}}\boldsymbol{+}\boldsymbol{64}\boldsymbol{.}\boldsymbol{54} \boldsymbol{X}_{\boldsymbol{1}} \boldsymbol{X}_{\boldsymbol{3}}\boldsymbol{-}\boldsymbol{23}\boldsymbol{.}\boldsymbol{07} \boldsymbol{X}_{\boldsymbol{2}} \boldsymbol{X}_{\boldsymbol{3}}$ | | | | | | |  |
| **Supplementary Table 2.** ANOVA table for the quadratic model calculated for [C8MIm] Cl (continues). | | | | | | | |  |
|  |  |  | |  | |  | |  |
|  |  |  | |  | |  | |  |
|  |  |  | |  | |  | |  |
| **Hyperoside (mg kg^-1^)** | | |  | |  |  |  |  |
| Model | 1283700 | | 9 | | 142640.00 | 369.19 | 1.62 x 10^-6^ |  |
| Linear | 3 | | 1086712 | | 362237 | 937.48 | <0.001 |  |
| X_1_ | 1 | | 1080641 | | 1080641 | 2796.73 | <0.001 |  |
| X_2_ | 1 | | 4724 | | 4724 | 12.23 | 0.017 |  |
| X_3_ | 1 | | 1346 | | 1346 | 3.48 | 0.121 |  |
| Square | 3 | | 189350 | | 63117 | 163.35 | <0.001 |  |
| X_1_*X_1_ | 1 | | 188771 | | 188771 | 488.55 | <0.001 |  |
| X_2_*X_2_ | 1 | | 2378 | | 2378 | 6.16 | 0.056 |  |
| X_3_*X_3_ | 1 | | 260 | | 260 | 0.67 | 0.449 |  |
| 2-Way Interaction | 3 | | 7656 | | 2552 | 6.6 | 0.034 |  |
| X_1_*X_2_ | 1 | | 740 | | 740 | 1.92 | 0.225 |  |
| X_1_*X_3_ | 1 | | 6711 | | 6711 | 17.37 | 0.009 |  |
| X_2_*X_3_ | 1 | | 205 | | 205 | 0.53 | 0.499 |  |
| Residual | 1931.80 | | 5 | | 386.35 | 4.77 |  |  |
| Total | 1285600 | | 14 | | 91832.00 |  |  |  |
| Pure error | 7.78 | | 2 | | 3.89 | 164.86 | 0.006 |  |
| Lack of fit | 1924.00 | | 3 | | 641.32 | 19.16 |  |  |
| R² | 0.999 | | 0.999 | |  |  |  |  |
| R² (máx) | 1.000 | | 1.000 | |  |  |  |  |
| **Model Equation** | $\boldsymbol{Y}\boldsymbol{=}\boldsymbol{736}\boldsymbol{.}\boldsymbol{72}\boldsymbol{+}\boldsymbol{367}\boldsymbol{.}\boldsymbol{53} \boldsymbol{X}_{\boldsymbol{1}}\boldsymbol{+}\boldsymbol{24}\boldsymbol{.}\boldsymbol{30} \boldsymbol{X}_{\boldsymbol{2}}\boldsymbol{+}\boldsymbol{12}\boldsymbol{.}\boldsymbol{97} \boldsymbol{X}_{\boldsymbol{3}}\boldsymbol{-}\boldsymbol{2}\boldsymbol{26.11}{\boldsymbol{X}_{\boldsymbol{1}}}^{\boldsymbol{2}}\boldsymbol{-}\boldsymbol{25}\boldsymbol{.}\boldsymbol{38} {\boldsymbol{X}_{\boldsymbol{2}}}^{\boldsymbol{2}}\boldsymbol{-}\boldsymbol{8}\boldsymbol{.}\boldsymbol{40} {\boldsymbol{X}_{\boldsymbol{3}}}^{\boldsymbol{2}}\boldsymbol{+}\boldsymbol{13}\boldsymbol{.}\boldsymbol{61} \boldsymbol{X}_{\boldsymbol{1}} \boldsymbol{X}_{\boldsymbol{2}}\boldsymbol{+}\boldsymbol{40}\boldsymbol{.}\boldsymbol{96} \boldsymbol{X}_{\boldsymbol{1}} \boldsymbol{X}_{\boldsymbol{3}}\boldsymbol{+ 7.15}\boldsymbol{X}_{\boldsymbol{2}} \boldsymbol{X}_{\boldsymbol{3}}$ | | | | | | |  |

**Supplementary Table 2.** (cont.) ANOVA table for the quadratic model calculated for [C8MIm] Cl.

| Source | Sum of squares | Degrees of freedom | Mean of square | *F* value | *p* value |  |
| --- | --- | --- | --- | --- | --- | --- |
|  |  |  |  |  |  |  |
| **Mangiferin (mg kg^-1^)** | |  |  |  |  |  |
| Model | 127860.00 | 9.00 | 14207.00 | 73.83 | 8.81 x 10^-5^ |  |
| Linear | 3 | 119257 | 39752 | 206.61 | <0.001 |  |
| X_1_ | 1 | 118027 | 118027 | 613.44 | <0.001 |  |
| X_2_ | 1 | 267 | 267 | 1.39 | 0.292 |  |
| X_3_ | 1 | 963 | 963 | 5 | 0.076 |  |
| Square | 3 | 5770 | 1923 | 10 | 0.015 |  |
| X_1_*X_1_ | 1 | 3131 | 3131 | 16.27 | 0.010 |  |
| X_2_*X_2_ | 1 | 1514 | 1514 | 7.87 | 0.038 |  |
| X_3_*X_3_ | 1 | 687 | 687 | 3.57 | 0.117 |  |
| 2-Way Interaction | 3 | 2831 | 944 | 4.9 | 0.060 |  |
| X_1_*X_2_ | 1 | 122 | 122 | 0.63 | 0.462 |  |
| X_1_*X_3_ | 1 | 2169 | 2169 | 11.27 | 0.020 |  |
| X_2_*X_3_ | 1 | 541 | 541 | 2.81 | 0.155 |  |
| Residual | 962.10 | 5.00 | 192.42 |  |  |  |
| Total | 128820.00 | 14.00 | 9201.50 |  |  |  |
| Pure error | 4.94 | 2.00 | 2.47 | 129.17 | 0.008 |  |
| Lack of fit | 957.16 | 3.00 | 319.05 |  |  |  |
| R² | 0.993 | 0.996 |  |  |  |  |
| R² (máx) | 1.000 | 1.000 |  |  |  |  |
| **Model Equation** | $\boldsymbol{Y}\boldsymbol{=}\boldsymbol{298}\boldsymbol{.}\boldsymbol{61}\boldsymbol{+}\boldsymbol{121}\boldsymbol{.}\boldsymbol{46} \boldsymbol{X}_{\boldsymbol{1}}\boldsymbol{-}\boldsymbol{5}\boldsymbol{.}\boldsymbol{77} \boldsymbol{X}_{\boldsymbol{2}}\boldsymbol{-}\boldsymbol{10}\boldsymbol{.}\boldsymbol{97} \boldsymbol{X}_{\boldsymbol{3}}\boldsymbol{-}\boldsymbol{29}\boldsymbol{.}\boldsymbol{12} {\boldsymbol{X}_{\boldsymbol{1}}}^{\boldsymbol{2}}\boldsymbol{+}\boldsymbol{20}\boldsymbol{.}\boldsymbol{25} {\boldsymbol{X}_{\boldsymbol{2}}}^{\boldsymbol{2}}\boldsymbol{+}\boldsymbol{13}\boldsymbol{.}\boldsymbol{64} {\boldsymbol{X}_{\boldsymbol{3}}}^{\boldsymbol{2}}\boldsymbol{+ 5.52}\boldsymbol{X}_{\boldsymbol{1}} \boldsymbol{X}_{\boldsymbol{2}}\boldsymbol{+ 23.29}\boldsymbol{X}_{\boldsymbol{1}} \boldsymbol{X}_{\boldsymbol{3}}\boldsymbol{+ 11.63}\boldsymbol{X}_{\boldsymbol{2}} \boldsymbol{X}_{\boldsymbol{3}}$ | | | | |  |
| **Supplementary Table 3.** ANOVA table for the quadratic model for choline acetate (continues). | | | | | |  |
| **Hyperoside (mg kg^-1^)** | |  |  |  |  |  |
| Model | 210020 | 9.00 | 23336.00 | 61.73 | 1.37 x 10^-4^ |  |
| Linear | 3 | 205163 | 68388 | 180.87 | <0.001 |  |
| X_1_ | 1 | 204236 | 204236 | 540.15 | <0.001 |  |
| X_2_ | 1 | 63 | 63 | 0.17 | 0.701 |  |
| X_3_ | 1 | 864 | 864 | 2.29 | 0.191 |  |
| Square | 3 | 3176 | 1059 | 2.8 | 0.148 |  |
| X_1_*X_1_ | 1 | 2464 | 2464 | 6.52 | 0.051 |  |
| X_2_*X_2_ | 1 | 1 | 1 | 0 | 0.968 |  |
| X_3_*X_3_ | 1 | 898 | 898 | 2.37 | 0.184 |  |
| 2-Way Interaction | 3 | 1678 | 559 | 1.48 | 0.327 |  |
| X_1_*X_2_ | 1 | 544 | 544 | 1.44 | 0.284 |  |
| X_1_*X_3_ | 1 | 571 | 571 | 1.51 | 0.274 |  |
| X_2_*X_3_ | 1 | 563 | 563 | 1.49 | 0.277 |  |
| Residual | 1890.30 | 5.00 | 378.06 |  |  |  |
| Total | 211910 | 14.00 | 15137.00 |  |  |  |
| Pure error | 2.89 | 2.00 | 1.45 | 435.39 | 0.002 |  |
| Lack of fit | 1887.40 | 3.00 | 629.14 |  |  |  |
| R² | 0.991 | 0.996 |  |  |  |  |
| R² (máx) | 1.000 | 1.000 |  |  |  |  |
| **Model Equation** | $\boldsymbol{Y}\boldsymbol{=}\boldsymbol{153}\boldsymbol{.}\boldsymbol{04}\boldsymbol{+}\boldsymbol{159}\boldsymbol{.}\boldsymbol{78} \boldsymbol{X}_{\boldsymbol{1}}\boldsymbol{+ 2.80}\boldsymbol{X}_{\boldsymbol{2}}\boldsymbol{-}\boldsymbol{10}\boldsymbol{.}\boldsymbol{40} \boldsymbol{X}_{\boldsymbol{3}}\boldsymbol{+}\boldsymbol{25}\boldsymbol{.}\boldsymbol{83} {\boldsymbol{X}_{\boldsymbol{1}}}^{\boldsymbol{2}}\boldsymbol{+}\boldsymbol{0}\boldsymbol{.}\boldsymbol{42} {\boldsymbol{X}_{\boldsymbol{2}}}^{\boldsymbol{2}}\boldsymbol{+ 15.59}{\boldsymbol{X}_{\boldsymbol{3}}}^{\boldsymbol{2}}\boldsymbol{+}\boldsymbol{11}\boldsymbol{.}\boldsymbol{66} \boldsymbol{X}_{\boldsymbol{1}} \boldsymbol{X}_{\boldsymbol{2}}\boldsymbol{-}\boldsymbol{11}\boldsymbol{.}\boldsymbol{95} \boldsymbol{X}_{\boldsymbol{1}} \boldsymbol{X}_{\boldsymbol{3}}\boldsymbol{+ 11.86}\boldsymbol{X}_{\boldsymbol{2}} \boldsymbol{X}_{\boldsymbol{3}}$ | | | | |  |

**Supplementary Table 3.** (cont.) ANOVA table for the quadratic model calculated for choline acetate.


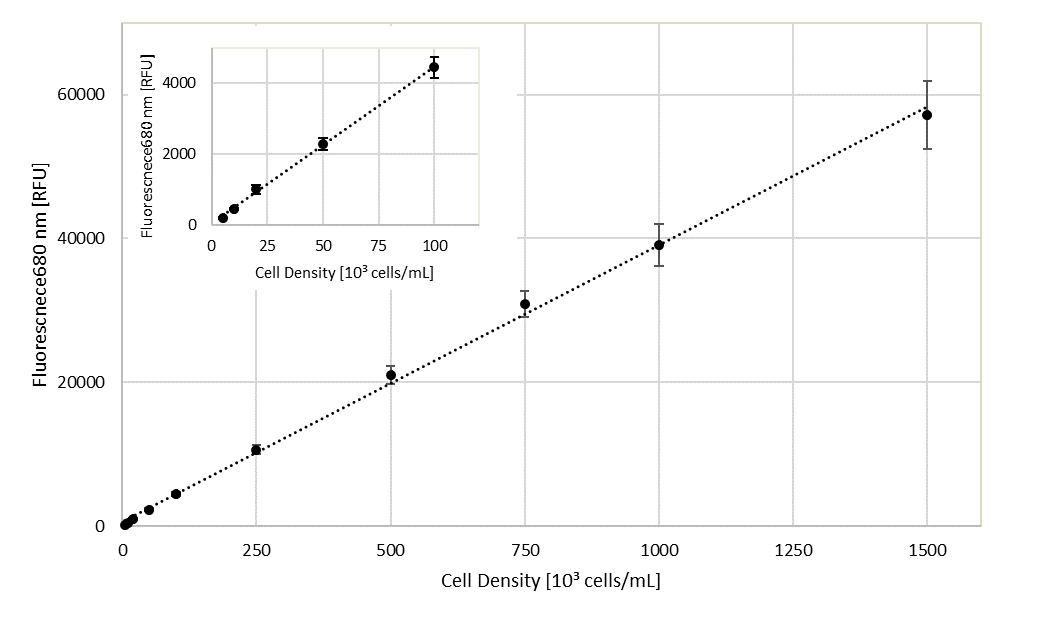


**Supplementary Figure 1.** Calibration curve to determine cell density. Amplification in left upper corner shows the calibration range used to determine low cell densities and limit of detection and quantification, n=3.


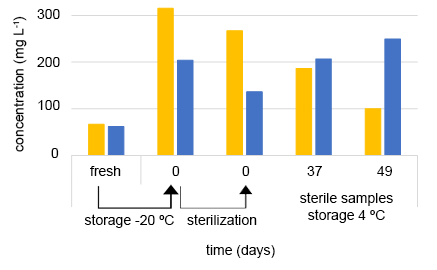


**Supplementary Figure 2.** Concentration variation of Mangiferin (yellow) and Hyperoside (blue) in the choline acetate extract during storage. The extract was sterilized by filtration for utilization in the algae growth inhibition test.


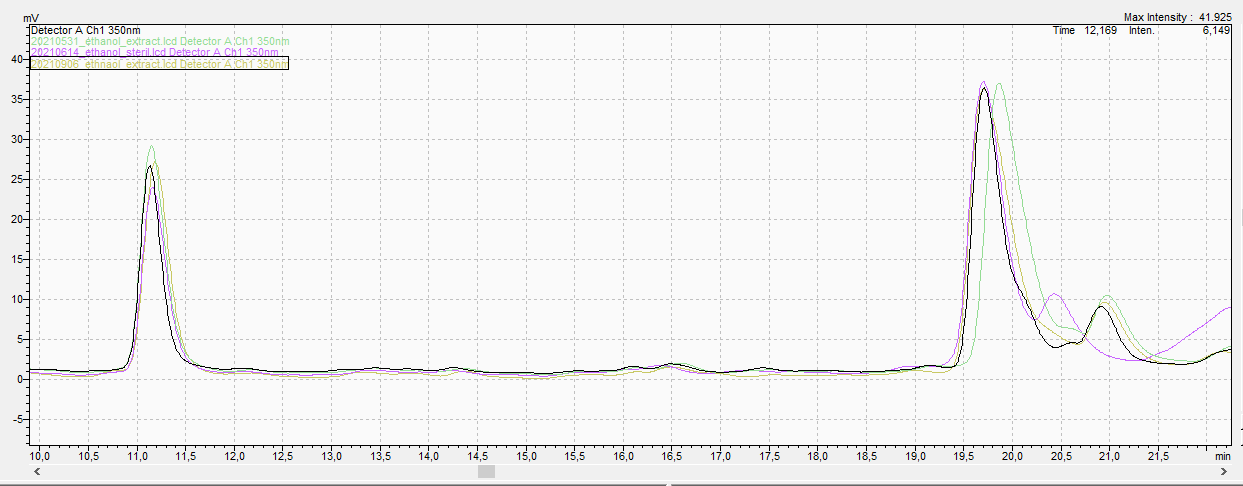


**Supplementary Figure 3.** Chromatograms of the ethanol/water mango processing waste extract after extraction (black line); unfrozen after 6-months storage at -20 °C (green line); after sterilization by filtration (pink line); and after 6-months unfrozen followed by 3-months storage in refrigerator at 4 °C (yellow line). Retention time for mangiferin peak is found at 11.2 min and hyperoside at 19.5 min.
